# Herpetofaunal assemblages of a lowland broadleaf forest, an overgrown orchard forest and a lime orchard in Stann Creek, Belize

**DOI:** 10.3897/zookeys.707.14029

**Published:** 2017-10-11

**Authors:** Russell Gray, Colin T. Strine

**Affiliations:** 1 Toucan Ridge Ecology & Education Society, 27.5 Hummingbird Highway, Stann Creek District, Belize; 2 Suranaree University of Technology, 111, Thanon Maha Witthayalai, Suranari, Mueang Nakhon Ratchasima District, Nakhon Ratchasima 30000, Thailand

**Keywords:** Amphibians, funnel-traps, human-altered habitats, passive-trapping, reptiles, lowland broadleaf forest

## Abstract

Understanding and monitoring ecological impacts of the expanding agricultural industry in Belize is an important step in conservation action. To compare possible alterations in herpetofaunal communities due to these anthropogenic changes, trapping arrays were set in a manicured orchard, a reclaimed orchard and a lowland broadleaf forest in Stann Creek district at Toucan Ridge Ecology and Education Society (TREES). Trapping efforts were carried out during the rainy season, from June to September, 2016, during which time the study site was hit by a category one hurricane between sampling sessions. Trapping yielded 197 individual herpetofauna and 40 different species overall; 108 reptile captures (30 species) and 88 amphibian captures (ten species). Reptiles and amphibians were more abundant in the lowland broadleaf forest and the manicured orchard area. Amphibian species diversity was relatively similar in each habitat type. Reptile captures were most diverse in the Overgrown Orchard Forest (OGF) and Overgrown Orchard Riparian Forest (OGR) and least diverse in the Lowland Broadleaf Forest (LBF). The findings of this study suggest that reptile and amphibian sensitivity to anthropogenically altered areas is minimal when enveloped by natural habitat buffers, and additionally, that extreme weather events have little impact on herpetofauna communities in the area.

## Introduction

Negative effects of agricultural development are well known for a number of taxa across the neotropics (Harvey et al. 2006; Offerman et al. 1995; Saab and Petit 1992). However, there is still contention regarding the impacts on herpetofauna and their assemblages (Lips et al. 2003; Suazo-Ortuno et al. 2008). Along with 391 reptiles and 307 amphibians endemic to the region, the Mesoamerica hotspot has remarkable herpetofauna species diversity in proportion to its surface area (Conservation International 2011; Mittermeier et al. 2011; Myers et al. 2000). According to the Mesoamerica Hotspot: Northern Mesoamerica Briefing Book (CEPF 2004), the Mesomerican hotspot is ranked first in reptile and second in amphibian species diversity when compared to other biodiversity hotspots around the world. Belize encompasses a great deal of undisturbed natural forest areas (Redo et al. 2012). However, due to lack of preventative legal framework and rising poverty rate, the country has been experiencing high rates of deforestation and large-scale sprawl of land for agricultural use (Young 2008). As a consequence of high deforestation rates, and its immense diversity of flora and fauna, Belize is an import part of the Mesoamerican biodiversity hotspot (Cherrington et al. 2012; DeClerck et al. 2010; Young 2008).

**Figure 1.**
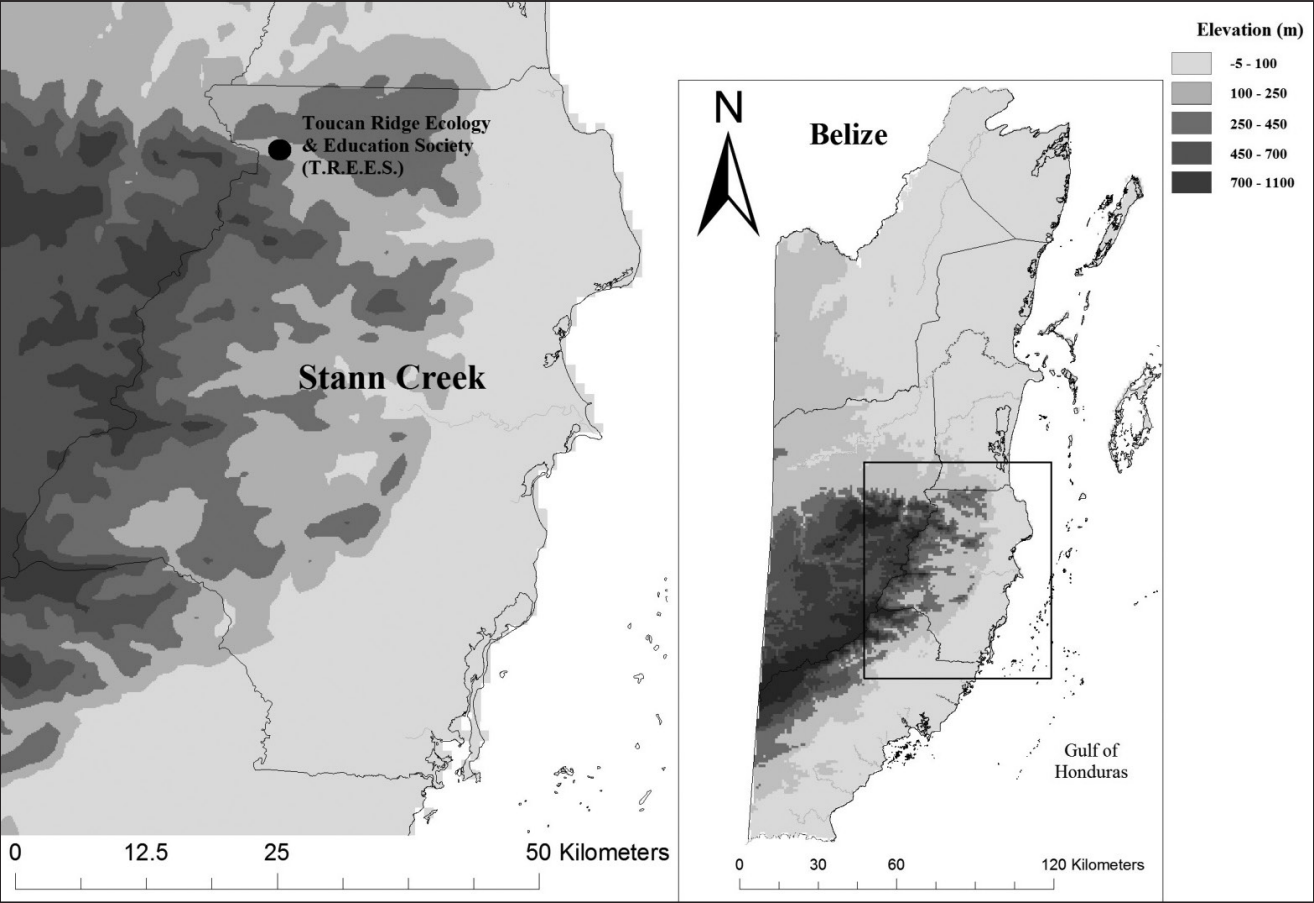
Stann Creek District, Belize, with the location of Toucan Ridge Ecology and Education Society (TREES).

The Stann Creek district of Belize (Fig. 1) is dominated by Mountain Pine Savannah and humid Lowland Broadleaf Forests, covered by granitic intrusions and limestone karst topography (Bridgewater 2012). Mountain Pine Savannah habitats are dominated by *Pinus oocarpa*, *Clusia* sp. and various grasses. Lowland Broadleaf Forest areas in Stann Creek, east of the Maya Mountains, are categorized as Class 3 Semi Evergreen Forests which are dominated by *Attalea cohune*, *Manilkara chicle*, *Pouteria reticulate*, *Terminalia amazonia*, *Bursera simaruba*, Eyma and *Brosimum alicastrum* (Penn et al. 2004). Montane habitats have a significant influence on biodiversity; the Maya Mountains extend southwest from the Stann Creek district of Belize into Guatemala and envelope 785,379 ha of the fourth key biodiversity area in the Selva Maya corridor (CEPF 2004). Limestone karsts, have been shown to have significant changes in diversity and species endemism, although are often overlooked for study (Brewer et al. 2003; Hughes 2017; Latinne et al. 2013). Flowing water bodies found throughout the district are fed by the Sibun River Watershed (SRW); the SRW drains the central portion of the country's water and empties into the Caribbean Sea (Boles 1999). Sprawling agricultural lands have reduced the forest cover of the Caribbean and Mesoamerican lowlands rapidly, attributed to a half-century of expansion from the Central American dry Pacific lowlands (Harvey et al. 2005; Pasos et al. 1994; Utting 1993). Forested areas of Belize are fragmented by approximately 19,424 ha of citrus orchard plantations, the majority of which are in the Stann Creek district (Smith 2016; Bridgewater 2012). Evidence suggests that agricultural and developmental land clearing can diminish forest-dwelling wildlife populations (Brownet al. 2014; Yue et al. 2015). Tropical forests are important in preserving wildlife assemblages and are very slow to regenerate to their original functions when cleared, if they return at all (Frost 1981; Gibson et al. 2011).

Biodiversity conservation is necessary in Central America and the whole of the neotropics in order to maintain ecosystem functions (Islebe et al. 2015; Spangler 2015). Furthermore, habitat loss driven by deforestation is a major driving factor for loss of ecosystem function (Hinsley et al. 2015). According to the study of seven Central American countries by Redo (2012), Belize was the highest ranking in woody vegetation cover (63%). However, deforestation in Belize between 2001 and 2014 was estimated to be 141,711 ha; a forest-cover decrease of 96.9% to 85.72% (Chicas et al. 2016; GFW 2017). With this increased deforestation, it is of paramount importance to monitor and evaluate effects on the fauna of Belize in order to develop counteractive conservation methods.

Five major ecological assemblages characterize Central American herpetofauna: 1) humid tropical, 2) arid tropical, 3) humid montane, 4) arid montane and 5) high montane; the humid tropical assemblages of lowland habitat areas contain highest species richness and endemism (Duellman 1966). After the global assessment, the status of Mesoamerican amphibians has been extensively evaluated (Stuart et al. 2004). Habitat loss has been correlated with 89% of threatened amphibian species; 52% of the 685 amphibian species in Mesoamerica (Young et al. 2004). Another major factor of amphibian decline is the chytrid fungal pathogen *Batrachochytrium dendrobatidis* which has been found to be prevalent in the Maya Mountains of Belize (Kaiser and Pollinger 2012). In comparison, the status of reptiles in Mesoamerica is less well-known (Johnson et al. 2015; Wilson et al. 2013). According to the Environmental Vulnerability Score (EVS) of Mesoamerican herpetofauna described by Johnson et al. (2015), amphibians have an average EVS of 14.7 and reptiles have an average EVS of 13.3; these scores indicate that amphibians are generally more vulnerable to habitat decline than reptiles, and overall, at a mid-range of vulnerability.

Therefore, the objective of this study was to monitor herpetofauna assemblages in forested areas and various anthropogenic altered areas in order to compare any possible differences in community structure. Comparisons of this nature test the hypothesis of whether or not agricultural land clearings reduce herpetofauna diversity and richness, and if so, whether or not reclamation of these habitats restores diversity and richness. An imperative facet of wildlife conservation is the understanding of how anthropogenic change affects fauna. It is with this understanding that proper conservation methods and mitigation techniques can be implemented.

## Materials and methods

**Figure 2.**
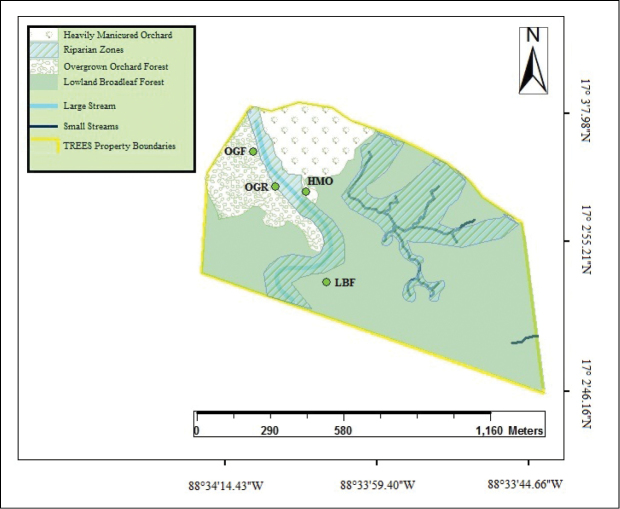
Toucan Ridge Ecology and Education Society (TREES) habitat map showing the Heavily Manicured Orchard (HMO), Overgrown Orchard Forest (OGF), Lowland Broadleaf Forest (LBF), and Overgrown Orchard Riparian Forest (OGR) areas, including locations where trapping arrays were set. Additionally, the map indicates the large stream which runs through the property and smaller streams that can be found throughout the forested areas.

***Study site.***—The study site was chosen at Toucan Ridge Ecology and Education Society (TREES) located between DMS; 17°03'07.98–17°02'46.16N, 88°34'14.43–88°33'44.66 W; WGS84 (Fig. 2). The TREES property encompasses approximately 200 acres (0.809 km^2^) of private land located at the foothills of the Maya Mountains. The dominant habitat on the property is lowland broadleaf forest, characteristic of the moist rainforest habitats commonly found throughout Belize (Stafford et al. 2010). The land also has an open and regularly manicured lime orchard which converges with the lowland broadleaf forest through a large area of overgrown citrus orchard, ecotone, and moist broadleaf riparian forest. A large stream, approx. 4–5 m in width, runs north to south (with some curvature) through the property, which is fed by several headwater streams from the Maya Mountains and floods regularly during heavy rains. Average rainfall for the study period was 7.04 mm during the day and 8.84 during mm during the night with the highest overnight rainfall during hurricane Earl (>150 mm).

***Site selection and herpetofaunal sampling.***—Herpetofaunal assemblages were assessed and monitored within four habitats using drift fence and funnel trapping systems. These study sites included: 1) a heavily manicured lime orchard (HMO), 2) an old orange orchard overgrowth forest (OGF), 3) an old orange orchard overgrowth forest with an adjacent riparian forest area (OGR) and 4) a virgin lowland broadleaf forest area disturbed only by walking trails (LBF). Both reclaimed orchard study sites have been undisturbed for approximately 15 years. Each habitat chosen for sampling in the study was identified and verified by knowledge of the current owners of the property, caretakers who have managed it since early 2000, and additionally by updated habitat analysis conducted over the span of three years (V. Kilburn and M. Charette, pers. comm. May 2015). We used previously conducted habitat analysis of the TREES property from former interns who used several plots with transect lines running through them in order to determine habitat diversity through Gap Analysis, using vegetation as key biodiversity elements.

Trapping arrays (one per study site) were constructed in convenient sample sites within the chosen study areas that conformed to three major requirements: 1) Besides the HMO, each end of drift fence arrays should be ≥10 m from any walking trails; 2) trapping arrays should be 30–40 m from the large stream running through the property and 3) the trapping arrays should be placed on relatively flat ground to prevent fence wings from being placed in a levy, valley or dip, in order to avoid flood risk which could result in mortality of trapped fauna.

The trapping array in the HMO was set in a secluded part of the orchard area (DDM; 17°03.144N, 088°34.148W; WGS84) at an elevation of approximately 177.394 m. This area was regularly manicured, though did not experience the same amount of human interference as other parts of the orchard as a transit and fruit harvesting area. This was an important factor to avoid human interference with traps. The OGF trapping system (DDM; 17°03.030N, 088°34.046W; WGS84) was set at an elevation of approximately 190.5 m, 10m away from a trail system that leads to the adjacent broadleaf forest area. The second trapping system in the overgrown orchard habitat (OGR) was set in a riparian zone approximately 119.9 m from the OGF trapping system (DDM; 17°03.050N, 88°34.138W; WGS84) at an elevation of 186.8 m. The Lowland Broadleaf Forest trapping array was set deep into the heavily forested area (DDM; 17°03.028N, 88°34.044W; WGS84) at an elevation of 192.0 m.

In order to cover sufficient surface area for trapping herpetofauna, each of the fences were set up in a ‘Y’ shaped formation, with the one wing of the ‘Y’ facing west, one facing south east and the other wing extending to the north west, the length of the formation running perpendicular to the nearby stream. Each wing of the array was 10 m in length and approximately 1 m high, relative to the terrain it crosses; the bottom of the fence were buried approximately 10–15 cm to reduce chances of fossorial and sub-fossorial herpetofauna from evading capture. Fencing material was 1.2 m black nylon mesh material which was supported by bamboo stakes set at every meter from the vertex of the array; bamboo stakes were cut at approximately 1.4 m in length to allow for the 1 m height to be maintained when set in the ground and buried; nylon fencing material was attached and secured to the bamboo via zip-ties and staples. Each of the four drift fence arrays were set with 12 funnel traps. Outer trap openings, on the ends of the drift fence, faced inward toward the vertex of the array and the inner trap openings faced away from the vertex. Traps were double-funnel and assembled using aluminium mesh screen material, with a 60 cm long entry chamber and a 70 cm long holding chamber; chamber height and width was approximately 30×30cm. Wire and staples were used to hold shape and secure the canters and ends of the traps. Funnel openings were approximately 6-8 cm in diameter and were fitted with a flap to reduce capture escape probability. Trap ends were secured with removable wire for convenient fauna release. All traps had the front lip buried in the soil to reduce trap avoidances; traps within HMO area or open canopy areas of the OGF and OGR were covered with a white sheet to prevent direct sunlight and overheating of trapped fauna and reduce risk of stress and fatality. Traps and fence damage issues were repaired and addressed on a same-day basis when needed. Trapping sample sessions ran for 14 days each month of the rainy season between June and late September, 2016 for a total of eight weeks of sampling. These dates were chosen in order to capture and assess herpetofauna during their most active period of the year. According to the historical temperature and rainfall averages recorded between 1991 and 2015 on The Climate Change Knowledge Portal by the World Bank Group (no date), the overall climate of the study period was a standard representative of previous years, and can therefore accurately represent data extrapolations.

***Environmental variables.***—In order to better understand the chosen study sites and the area surrounding the trapping systems, habitat variables were taken into account. We conducted a micro/macro-habitat analysis at each study site. Macro-habitat data was collected by measuring and forming a 10×10 m quadrat at the end of each wing of the drift fence array with colored flagging tape. Once the quadrats were established, several participating assistants walked, at a full arm’s length apart from each other through the quadrat and collected plants to be identified in order to find the dominant vegetative species in the habitat area. Plant identification was verified with the assistance of Belizean botanist David Tzul using the Checklist of the Vascular Plants of Belize, with Common Names and Uses (Balick 2000) and Trees of Belize (Harris 2009).

Microhabitat data was collected at each of the four study sites to evaluate understory vegetation, ground cover/composition and canopy cover. A 1×1m quadrat divided into four equal sub-sections was used to evaluate understory vertical vegetation density, leaf/grass cover and composition, and canopy cover. The quadrat was placed beside the 12 traps of each array. To evaluate standing vegetation, a 2m measuring pole was placed in the centres of the four subsections in the quadrat and touch points were tallied within every 20 cm (Valentine et al. 2012: Table 1). We used a visual estimate of the four subsections for leaf litter percentage. Leaf litter and grass cover was calculated by pushing a 30 cm ruler in each subsection until solid ground was felt, and the four recorded values (± 0.5 cm) were averaged. A spherical handheld crown densitometer was used at each side of the quadrat to derive an average canopy cover percentage. Microhabitat analysis was conducted before and after Hurricane Earl to assess any possible significant changes to the immediate study area.

**Table 1.** Comparison of microhabitat variables averaged from 12 points at each sampling site pre/post Hurricane Earl (note: HMO grass is regularly manicured and not constant, therefore it is ranked “-” on the chart for litter (%) and depth analysis; vertical vegetation density was factored in with exceptions (≤ 20 cm)); Before (B) and After (A) vertical vegetation values are shown side-by-side in the table.

Microhabitat variable	HMO		OGF		OGR		LBF	
	Mean	SD (±)	Mean	SD (±)	Mean	SD (±)	Mean	SD (±)
Canopy cover (%) pre	23	30	91	4	77	24	98	1
Canopy cover (%) post	12	12	44	27	13	28	95	3
Leaf Litter (%) pre	-	-	59	22	51	30	56	25
Leaf Litter (%) post	-	-	87	12	87	12	75	28
Litter depth (cm) pre	-	-	7.02	60	4.90	2.24	2.98	1.61
Litter depth (cm) post	-	-	2.69	95	1.71	1.42	2.05	1.36
Vertical Vegetation Density (Number of touch points)								
0–20 cm(B:A)	-	-	1.75:0.17	4.94:0.58	0.75:0.08	1.76:0.29	3.91:0.58	3.5:0.67
20–40 cm(B:A)	0.58:0.08	1.16:0.29	1.33:1.25	3.70:2.93	0.08:0.5	0.29:0.67	0.5:0.5	1:0.90
40–60 cm(B:A)	0.08:0	0.29:0	0.43:0.08	0.67:0.29	0.33:0.58	0.78:1.16	0:0.08	0:0.28
60–80 cm(B:A)	0.08:0.08	0.29:0.28	0.25:0.08	0.62:0.29	0.08:0.17	0.29:0.39	0:0.25	0:0.62
80–100 cm(B:A)	1.67:0.33	0.58:0.78	0.17:0.08	0.58:0.29	0.41:0	0.67:0	0:0.08	0:0.29
100–150 cm(B:A)	0.67:0.42	1.62:1.0	0.5:0.5	0.80:1	0.75:0.58	0.97:0.79	0.5:0.58	1.24:0.90
150–200 cm (B:A)	0.45:0.08	1.44:0.29	1.5:0.5	2.39:0.67	1.08:0.5	1.31:1.24	1.08:0.25	1.44:0.62

As another measure of environmental variable analysis, we took general micro-climate data (temperature and Relative Humidity [RH]) at each plotted site every day during trap checks (between 0800h and 1000h) using a HTC-1 temperature and humidity meter (Temperature Accuracy: Â ± 1°C; Humidity Range :10-99%; RH accuracy: 60% Â ± 5% RH). Along with this, we recorded rain data each day at 0800h and 2000h from a plastic rain gauge set in an open area.

***Data analysis.***—We performed all statistical analyses in RStudio V. 0.99.903 using packages “vegan” and “BiodiversityR” (Kindt and Coe 2005; Oksansen et al. 2016; R Core Development Team 2016); we created maps using ArcMapper V10.4; basemaps and other map datum for Belize were obtained from the Biodiversity & Environmental Resource Data System (BERDS) website (Meerman and Clabaugh 2016). We used the field guides “A Field Guide to the Amphibians and Reptiles of the Mayan World: The Lowlands of Mexico, Northern Guatemala, and Belize” by Julian C. Lee and “Amphibians and Reptiles of Northern Guatemala, the Yucatan, and Belize” by Jonathan A. Campbell to identify herpetofauna, when necessary; Identifications were further verified by the publication on their holotype and most recently updated taxonomic papers (AmphibiaWeb 2016; IUCN 2016; Uetz n.d.). We added analysis of Hurricane Earl; however, the capture data was not significantly different between reptiles and amphibians caught before and after the hurricane, so the focus remains on the habitat variations with the hurricane included as an important variable, as there are no comparative data from previous years of herpetofauna trapping in this area. We compared canopy changes before and after the hurricane using a Wilcoxon signed-rank test, and performed other habitat comparisons by averages. Herpetofauna species capture data were analysed to compare any trends or patterns in species capture rates, species richness and species diversity between the different study sites. Amphibian and reptile community assemblages and capture data were analysed separately, as their reactions and sensitivity to habitats affected by anthropogenic change can be disparate and the capture rates between them in the study were significantly different in all habitat types. Reptile and amphibian capture rates were compared between all habitat types using a Kruskal-Wallace ranked sum test, and a post-hoc Wilcoxon signed-rank test to compare habitats that appeared significantly different after forming boxplots. An ANOVA was used to test for overall variations in species diversity. For all statistical analysis tests, α = 0.05. Numbers of individuals and species were evaluated at each site with a calculated Shannon-Weiner diversity index (Krebs 1989). Further analysis was conducted to quantify extrapolated richness values for unseen reptile species.

## Results

***Habitat variation.***—Habitat variations were recorded using the micro/macro-habitat analysis for each of the four habitat areas studied:

*Heavily Manicured Orchard.*—The HMO had an average temperature of 31.15°C and an average RH of 76.2% throughout the duration of the study. The soil was relatively dry in comparison to the other plotted areas and was covered in grass rather than leaf litter as groundcover, the open area was thinly spotted with *Citrus aurantifolia* lime trees covered in water bearing *Aechmea* sp. bromeliads. There were no canopies over the trapping systems; however there were dense canopy edges (> 2 m) on the west and north-west wings. After the hurricane, large trees fell over top of the north-west wing providing a heavily shaded canopy area over two of the traps.

*Overgrown Orchard Forest.*—The OGF habitat area had an average temperature of 27.45 °C and average RH of 84.1% throughout the duration of the study. The ground of this site was covered in leaf litter and moist soil which turns to soft mud after rains. Vegetation consisted of small scattered bryophytes and lycophytes with dominant tree species being *Cupania* sp. The site was covered by relatively heavy canopy with an extensive liana complex extending from tree to tree. Following the hurricane, the canopy cover decreased and tangled liana complexes hung large concentrations of vegetation over the centre and west wing of the trapping system.

*Overgrown Orchard/Riparian Forest.*—The vegetation, soil composition and canopy cover in the OGR were relatively similar to the OGF habitat with the exception of a small wetland area, composed of a thick patch *Costus* sp. and shallow muddy water approximately 15-25 cm in depth, approximately >5m from the end of the west wing and extending further past the 10×10 quadrat area documented. The site had an average temperature of 26.71 and average humidity of 84.3% throughout the study. After the hurricane, the canopy cover was significantly altered with nearly all vegetation falling away from the trapping array and none overhanging, as has occurred in the other sites.

*Lowland Broadleaf Forest.*—The canopy cover of the LBF was very heavy; soil was moist; ground cover consisted of leaf litter, scattered bryophytes, lycophytes and many sapling trees. Scattered *Bactris major* and large *Attalea cohune* were surrounding the trapping system; the dominant tree species were *Xylopia* sp., *Hirtella Americana*, and *Vochisia hondurensis*. The site had an average temperature was 26.89 °C and RH was 83.2% throughout the study. After the study, there was very little alteration to the vegetation in the study site, assumedly the thick vegetation levels inhibited and broke down the heavy winds.

**Figure 3.**
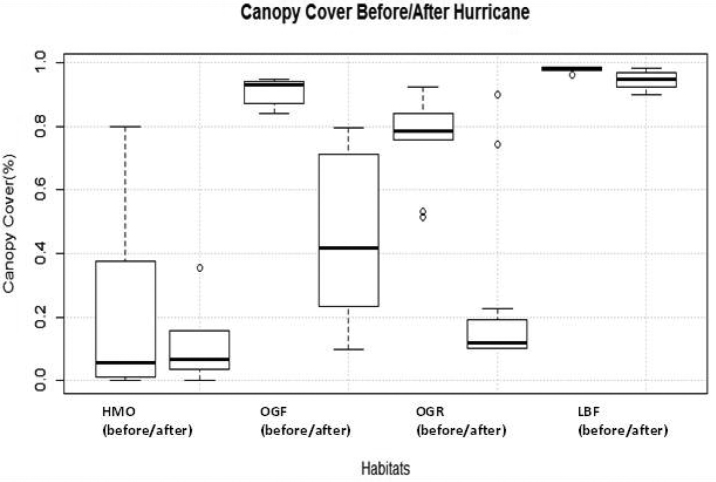
Boxplots representing the values (%) of canopy cover in each habitat taken before and after Hurricane Earl.

***Microhabitat variables pre/post hurricane.***—In order to show the difference in canopy changes from Hurricane Earl which occurred after two of the four sample sessions, we took canopy cover data before and after hurricane Earl (Fig. 3). The data shows that there were significant alterations in canopy percentages in the OGF (Wilcoxon signed-rank; V = 78, P = 0.002) and OGR (Wilcoxon signed-rank; V = 78, P = 0.002) and slight canopy reductions in the HMO (Wilcoxon signed-rank; V = 42, P = 0.423); there were no significant canopy alterations in the LBF (Wilcoxon signed-rank; V = 75, P = 0.005).

**Figure 4.**
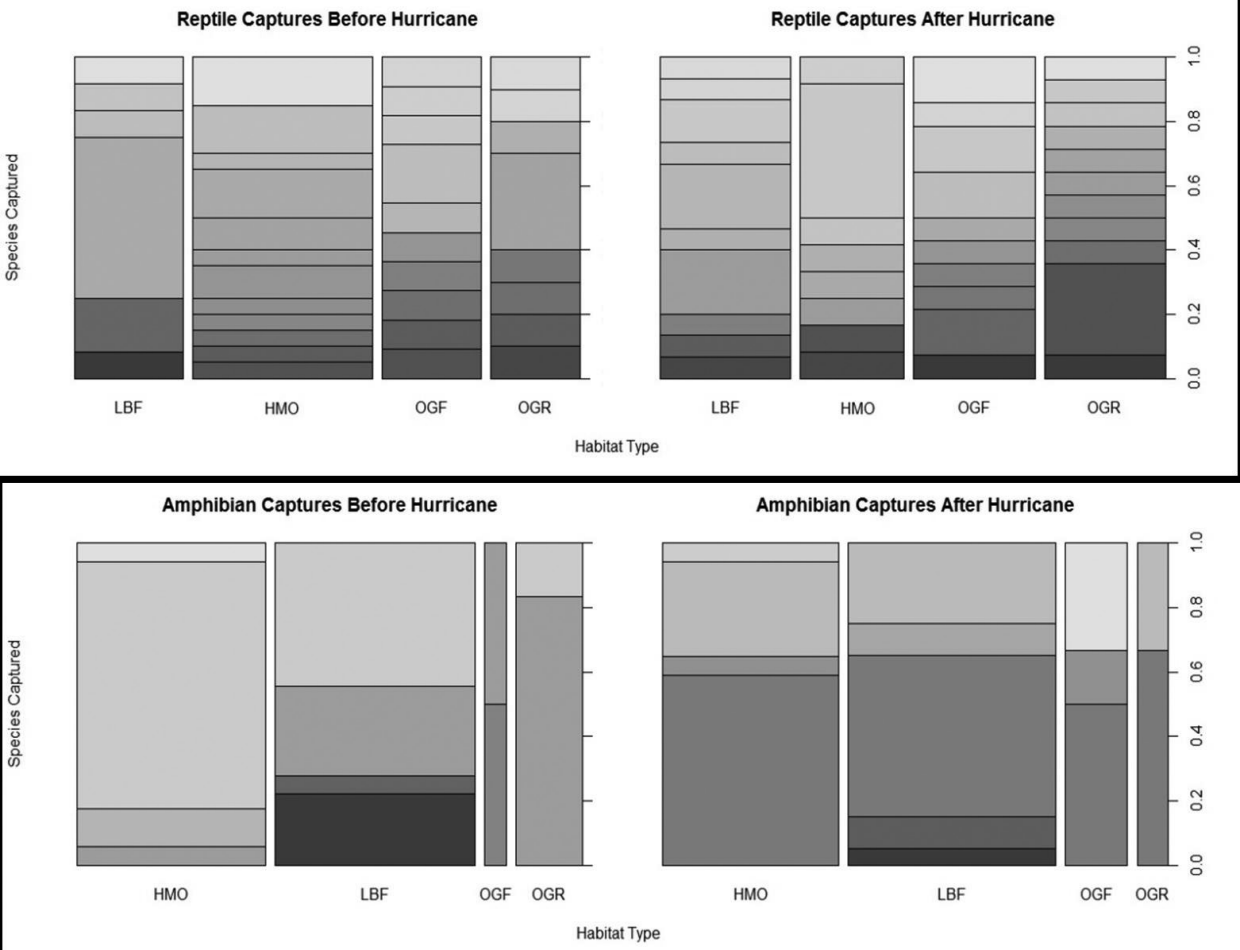
Bar graphs exhibiting species capture diversity and richness in reptiles and amphibians before and after Hurricane Earl.

***Pre/post hurricane species richness and diversity.***—The analysis of each habitat studied shows that although habitat areas were significantly altered after the hurricane there were no significant comparable differences in capture rates, species richness or diversity (Fig. 4). Reptiles caught before the hurricane yielded 23 species (53 individuals) and after yielded 23 species (55 individuals). Amphibian captures before the hurricane yielded 7 species (43 individuals, and after yielded 8 species (46 individuals).

Before the hurricane, the trapping system in the LBF yielded six species (12 individuals); HMO yielded 12 species (20 individuals); OGF yielded ten species (eleven individuals) and OGR yielded 8 species (10 individuals). Amphibian captures before the hurricane in the LBF yielded 4 species (18 individuals); HMO yielded 4 species (17 individuals); OGF yielded 2 species (2 individuals) and OGR yielded 2 species (6 individuals). Post-hurricane reptiles in the LBF yielded 10 species (15 individuals); HMO yielded 8 species (12 individuals); OGF yielded ten species (14 individuals) and OGR yielded 11 species (14 individuals). Post-hurricane amphibian captures in the LBF yielded 4 species (20 individuals); HMO yielded 4 species (17 individuals); OGF yielded 3 species (6 individuals) and OGR yielded 2 species (3 individuals).

***Capture rates.***—During this study, trapping efforts yielded 197 individual herpetofauna and 40 different species overall; 108 reptile captures (30 species) and 89 amphibian captures (ten species) (Table 2). Furthermore, traps captured 22 species unique to their particular habitat, 17 reptiles, and five amphibians.

**Table 2.** Amphibian and reptile pecies captured in each habitat type throughout the study.

Family	Species	Habitat type				
		HMO	OGF	OGR	LBF	Total
Ranidae	*Lithobates brownorum*	3	1			**4**
	*Lithobates juliani*				2	**2**
	*Lithobates vaillanti*	18		2	12	**32**
Craugastoridae	*Craugastor chac*				7	**7**
	*Craugastor sabrinus*		1			**1**
Bufonidae	*Incilius valliceps*	3	12	7	15	**37**
	*Rhinella horribilis*	1				**1**
Eleutherodactylidae	*Eleutherodactylus leprus*	1				**1**
Hylidae	*Smilisca baudinii*		2			**2**
Plethodontidae	*Bolitoglossa dofleini*				1	**1**
Kinosternidae	*Kinosternon leucostomum*			2		**2**
Corytophanidae	*Basiliscus vittatus*		1	2		**3**
Xantusiidae	*Lepidophyma flavimaculatum*		1		1	**2**
Dactyloidae	*Norops lemurinus*				4	**4**
Gekkonidae	*Coleonyx elegans*	2	1	5		**8**
Scincidae	*Scincella cherriei*	8	2	1	3	**14**
	*Marisora brachypoda*	1				**1**
Teiidae	*Holcosus undulatus*				1	**1**
Colubridae	*Coniophanes fissidens*				1	**1**
	*Coniophanes imperialis*				2	**2**
	*Drymarchon melanurus*		2	1		**3**
	*Drymobius margaritiferus*		1	1		**2**
	*Imantodes cenchoa*	1				**1**
	*Lampropeltis abnorma*		1			**1**
	*Leptodeira polysticta*	1				**1**
	*Leptophis ahaetulla*			1		**1**
	*Leptophis mexicanus*	2	1	1		**4**
	*Mastigodryas melanolomus*	1	1	1		**3**
	*Ninia diademata*	2	2			**4**
	*Ninia sebae*	4	2	1	2	**9**
	*Phrynonax poecilonotus*		3		1	**4**
	*Pliocercus elapoides*				1	**1**
	*Rhadinaea decorata*				1	**1**
	*Scaphiodontophis annulatus*		1			**1**
	*Sibon nebulatus*	1	1	2		**4**
	*Spilotes pullatus*	1				**1**
	*Tantilla hendersoni*		1		1	**2**
	*Tropidodipsas sartorii*			1		**1**
	*Xenodon rabdocephalus*		2	1		**3**
Viperidae	*Bothrops asper*	2	1		1	**4**
Elapidae	*Micrurus diastema*	2	1	3		**6**
	*Micrurus hippocrepis*	4		1	9	**14**
**Grand Totals**		**58**	**41**	**33**	**65**	**197**

**Figure 5.**
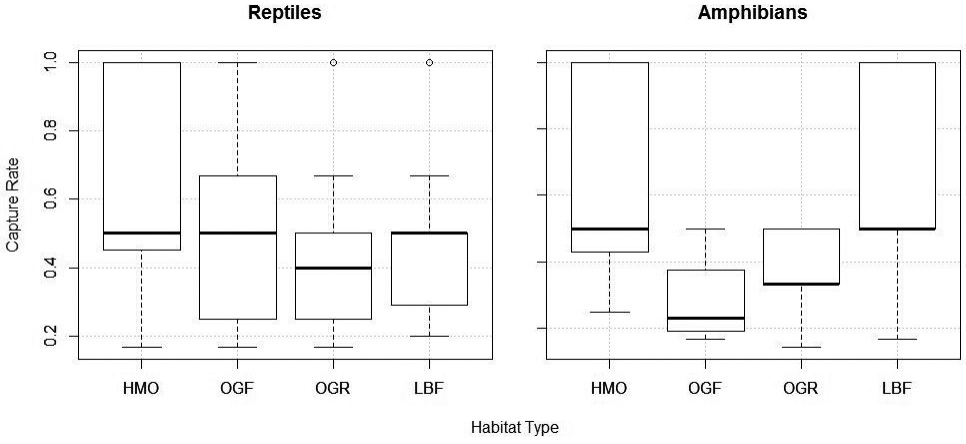
Boxplots of reptile and amphibian capture rates by trap night in the Heavily Manicured Orchard (HMO), Overgrown Orchard Forest (OGF), Overgrown Orchard/Riparian Forest, (OGR) and Lowland Broadleaf Forest (LBF).

Reptile and amphibian capture rates were analysed per plot-night for differences between habitat areas (Fig. 5). Amphibian capture rates per trap night were not significantly different between habitat types collectively (Kruskal-Wallis; χ^2^ = 6.0478, df = 3, *P* = 0.109); however, the capture rates in the OGF were significantly lower than both the HMO (Wilcoxon signed-rank; W = 8, P = 0.016) and LBF (Wilcoxon signed-rank; W = 14, P = 0.027). Although reptiles were captured in higher numbers than amphibians, there were no significant differences in capture rates collectively (Kruskal-Wallis; χ^2^ = 4.2267, df = 3, *P* = 0.238) or between habitats individually.

**Figure 6.**
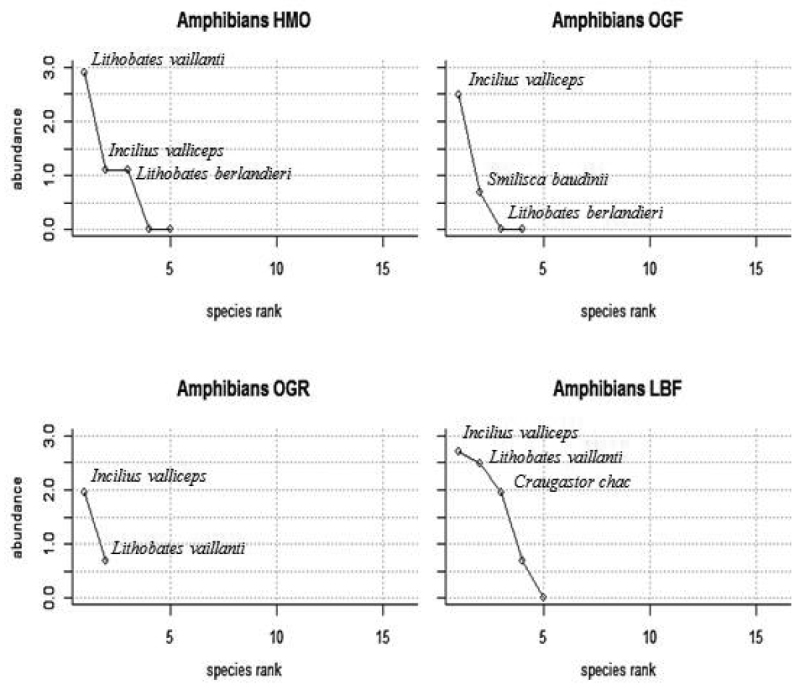
Amphibian rank abundance curves (using the logarithm of abundances) for the Heavily Manicured Orchard (HMO), Overgrown Orchard Forest (OGF), Overgrown Orchard Riparian Forest (OGR) and Lowland Broadleaf Forest (LBF); the three most abundant species captured in each site is labeled in each of the four plots.

**Figure 7.**
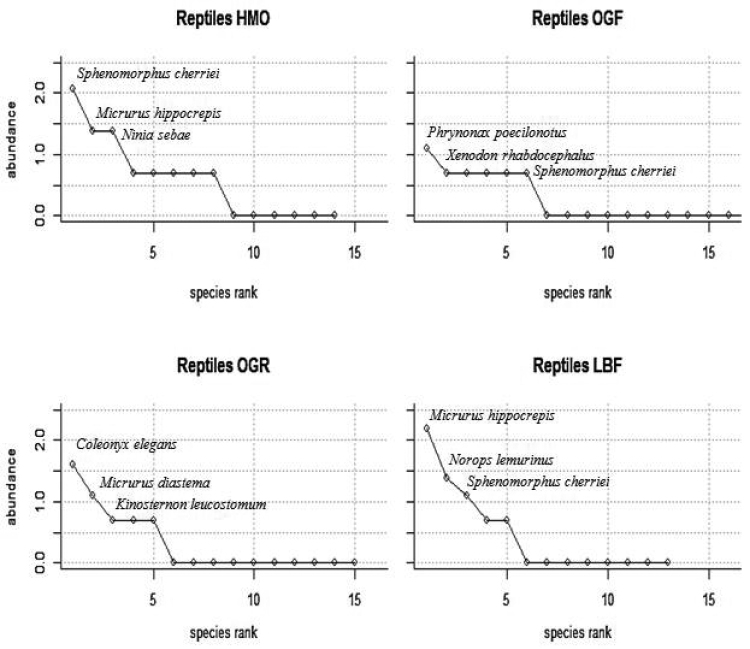
Reptile rank abundance curves (using the logarithm of abundances) for the Heavily Manicured Orchard (HMO), Overgrown Orchard Forest (OGF), Overgrown Orchard Riparian Forest (OGR) and Lowland Broadleaf Forest (LBF) study sites; the three most abundant species captured in each site is labeled in each of the four plots.

***Rank abundances*.**—Amphibian rank abundance curves between the four sites show no cogent species richness in amphibians (Fig. 6). Two species, *Incilius valliceps* and *Lithobates vaillanti*, had the highest ranking abundance in all habitats except OGF; the curves show that amphibian diversity and abundance is most prominent in LBF and least prominent in OGR; with OGF and HMO having relatively similar curves. Reptile rank abundance curves had relatively different slopes among the habitat types (Fig. 7). Although most habitats produced a low quantity of individuals captured, all of the plots have a relatively high number of species diversity; the lowest diversity habitat being HMO and the highest diversity habitat being OGF. When comparing these plots, it is evident that some species (i.e., *Scincella cherriei*) appear to be abundant in HMO, OGF, and LBF habitats, though the species is not present in the OGR habitat.

**Table 3.** Shannon-Wiener diversity indexes, means and standard deviations of reptiles and amphibians between each sample site are compared.

**Forest Type**	**Amphibians**			**Reptiles**		
	**Average observed species**	**Average Shannon Diversity**	**SD (±)**	**Average observed species**	**Average Shannon Diversity**	**SD (±)**
*Heavily Manicured Orchard*	2.6	1.00	5.54	0.5	3.26	0.63
*Overgrown Orchard Forest Overgrown*	1.6	0.82	3.71	0.46	2.97	0.69
*Orchard Riparian Forest*	0.9	0.52	2.23	0.43	2.83	0.68
*Lowland Broadleaf Forest*	3.70	1.30	5.64	0.48	3.07	0.66

**Figure 8.**
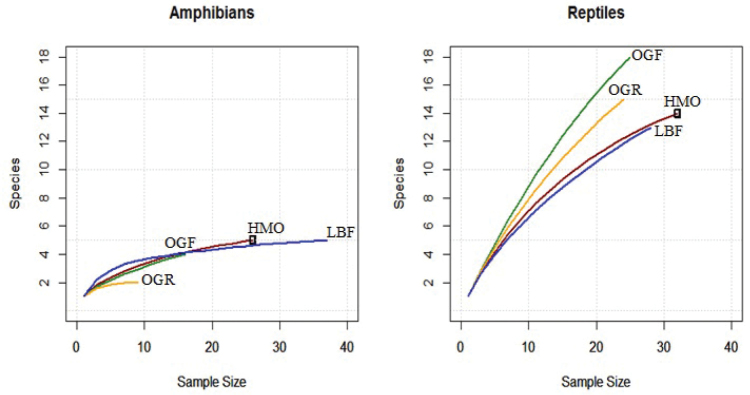
Rarefaction curves displaying sample based species richness and diversity for amphibian and reptiles captured in each habitat type.

***Species richness and diversity.***—Both species richness and diversity contrasted significantly between reptiles and amphibians in each habitat site (Fig. 8). Low levels of species richness and diversity was uniform for amphibians captured; curves show relatively similar slopes for HMO, OGF and LBF and indicate that species richness decreases from LBF, HMO, OGF to OGR in that respective order.

Reptiles had comparatively higher species richness and diversity in each habitat with HMO and LBF showing similarities in their richness, although HMO is slightly higher in diversity. Diversity is highest in OGF and lowest in LBF; richness is highest in HMO and lowest in OGR. The curves appear to indicate there is significant probability of discovering unseen species in their extrapolated richness values.

There were no significant differences in reptile diversity between each of the habitats studied, (ANOVA; F = 0.661, *P* = 0.869); similarly, amphibians too showed no significant difference in diversity of captured species in each habitat (ANOVA; F = 0.258, *P* = 0.854).

***Possibility of unseen species.***—We calculated values using the Chao 2 richness estimator and Abundance-based Coverage Estimation (A.C.E.) in order to estimate extrapolated richness values in each habitat for reptile species (Chao 1984; Chao et al. 2000; Colwell and Coddington 1994) (Table 4); Amphibian curves appeared to flatten out, indicating low probability of unseen species and were therefore omitted from the analysis.

**Table 4.** Extrapolated richness estimates evaluated through Chao1 index and Abundance-based Coverage Estimator (A.C.E.)

Forest Type	Reptiles				
	observed species	Chao1 Index	SE (±)	A.C.E.	SE (±)
*Heavily Manicured Orchard*	14	16.50	2.89	21.39	2.64
*Overgrown Orchard Forest Overgrown*	18	29.0	8.46	34.61	2.39
*Orchard Riparian Forest*	15	26.25	9.52	34.13	2.95
*Lowland Broadleaf Forest*	13	22.33	8.82	32.3	3.82

## Discussion

A total of 56 trap nights, spanning four months of the rainy season, yielded 40 species of herpetofauna (197 individuals); 108 reptile captures (30 species) and 88 amphibian captures (ten species). Trapping systems captured 49 frogs (seven species), 38 toads (two species), and only one species of salamander. Gulf coast toads *Incilius valliceps* and ranid frogs from the *Lithobates* genus were the most abundant amphibians captured (*Lithobates vaillanti* being the most abundant species); *Rhinella horribilis* (HMO), *Craugastor sabrinus* (OGF), *Eleutherodactylus leprus* (HMO) and *Bolitoglossa dofleini* (LBF) were the rarest, with only one individual of each species being caught in traps. In regards to reptiles, traps captured 74 snakes (25 species), 10 lizards (4 species), 8 geckos (1 species), 15 skinks (2 species) and two turtles (1 species).

Since the study sites were restricted to the TREES property, the trapping systems were all within close proximity to one another (μ = 267.6 m). In addition to this, there are several variations in habitat types; some are drastic (i.e., heavily manicured orchard areas bordering unaltered broadleaf forest areas), whereas some can be as miniscule as vegetation variations (i.e., lowland santamaria variant and lowland negrito-nargusta variant) within the broadleaf forest area (Penn et al. 2004). Habitat heterogeneity could result in increased species richness and diversity throughout the study areas (MacArthur and MacArthur 1961). However, this hypothesis must be factored in with the probability of decreased herpetofaunal species diversity due to open, human altered areas which lack significant shelter, such as woody debris (Whiles and Grubaugh 1996). It has been previously documented that herpetofauna species richness in agricultural areas with nearby strips of natural habitats can be greater than those without (Biaggini and Corti 2015). Natural broadleaf forest habitat strips were surrounding the trapping array in the HMO, possibly contributing to the species richness. This may indicate that small pockets of manicured land for agriculture may have reduced effects on herpetofauna communities so long as they have surrounding natural forest areas.

Herpetofaunal species diversity exhibited some extent of variation between the habitats studied; species richness was higher in reptiles than amphibians. Individual captures were highest in the LBF (n = 65) and HMO (n = 58) and lowest in the OGR (n = 33); diversity of species was highest in OGF (n=22) and relatively similar in HMO (n = 19), LBF (n = 18) and OGR (n = 17). Since this study only utilized standard Y-shaped funnel trapping system per habitat, incorporating pitfall traps and using different or modified funnel traps/drift fence configurations could increase trapping potential, particularly for anurans (Crosswhite 1999; Greenberg et al. 1994; Ribeiro Júnior et al. 2008). Moreover, including one or two more systems per habitat could increase capture rates and representativeness, providing better insight to herpetofaunal assemblages and species diversity; unfortunately, herpetological research in Belize is underfunded, so the study was performed under strict financial restraints. Conjointly, the study spanned through the rainy season, due to higher probability of capturing herpetofauna in what is generally their most active period. Nevertheless, if the study duration was extended throughout the year it may be a more acute representation of herpetofaunal assemblages in respects to seasonal variations, as some species may be more active at other times of the year (Todd et al. 2007). In addition to the sampling time constraint, the second trapping system set in the Overgrown Orchard Forest was set after the first sample session, providing a reduced capture capability; nevertheless, the trapped fauna accurately represented an abundant species diversity potential of the habitat areas so the data simply may not represent the richness potential individuals captured.

As this project was run for a single season, there are a number of cautions necessary for interpreting our dataset. The richness and abundance estimates may have been inflated or deflated by temporary boom and bust cycles of prey items during this particular season. It is also possible that we simply had an anomalous season and therefore recommend more extensive monitoring through a longer sampling period. Multi-year studies allow for monitoring survivorship of marked individuals, and trends in both activity and movement throughout the seasons, making them more robust. However, our study is intended as a snapshot view of the herpetofaunal communities within the different forest types of the study area in hopes to generate more interest for community level research within Belize, and to provide a baseline dataset from which to work.

The findings of this study can be used in conjunction with future herpetology and ecology work within the Belize in regards to community structure in anthropogenically-altered habitat areas. Monitoring efforts of herpetofauna in various habitats may assist in the creation of feasible conservation methods. Overall, there is now evidence of the effectiveness of drift fence and funnel trapping system use to monitor herpetofauna in Belize. Furthermore, the limitations of this study regarding lack of previous replicate studies, spatial autocorrelations, and changing environmental variables are understood.

The suitability of funnel traps in conjunction with drift fences is known to be an effective passive capture method for monitoring terrestrial herpetofauna (Dorcas and Williams 2009; Todd et al. 2007). Although these trapping systems may result in fewer captures, they promote more standardization of effort, which allows for more robust comparisons among different sites. Many methods in Belize have been utilized to study herpetofauna, although no published studies have included drift fences and funnel traps for passive trapping efforts. That being said, there have been efforts to use drift fences and pitfall traps to monitor other fauna, such as small mammals and amphibians (Engilis et al. 2012). Replicate studies using similar trapping methods should be implemented in the future for further verification of our findings.

Hurricane Earl significantly effected study site vegetation (i.e. canopy cover and standing vegetation), though didn’t significantly alter capture diversity. One possible result of post-hurricane habitat alterations were the captures of two individuals of *Tantilla hendersoni*, a data deficient species of centipede eating snake known from only one prior individual record (Hofmann et al. 2017; Wilson and Mata-Silva 2015). Both snakes were captured after the hurricane struck the study sites. *Tantilla* are known to be fossorial and cryptozoic snakes and their activity during the post-hurricane sample sessions may have been to relocate due to damaged habitat areas or deracination of prey items. Other interesting occurrence, were the captures of arboreal snake species *Imantodes cenchoa*, and *Leptodeira polysticta*, both in the HMO just a few days before the hurricane. This was the only instance of capture for both species, which brings to question whether the captures were coincidental or influenced by the snakes seeking shelter in the manicured area, away from falling trees.

Predated fauna may also account for species capture data to be reduced. R. Gray and A. Pelletier observed a *Micrurus hippocrepis* captured underneath a trap in the OGF, seemingly attempting to access the *Ninia sebae* captured within. This leads to the assumption that some herpetofauna trap-mates could potentially have been taken by other predatory herpetofauna, as many of the snake species caught are known to have diets consisting of lizards, skinks, frogs, toads and other snakes (Lee 2000; Campbell 1999). Due to the possibilities of trap mates being predated, data may lack some unique or rare species occurrences; however, the general trend of dominant species in each habitat would likely remain the same.

A study by McCoy (1970) in Middlesex (a village area near TREES) yielded similar information on snake species and their abundances in citrus plantations, *Ninia sebae* and *Micrurus hippocrepis* being found in higher abundances than other snakes. Our study suggests a similar trend, as the two most abundant species in the HMO were *N. sebae* and *M. hippocrepis*.

## Conclusions

Intensive trapping studies should be implemented throughout the year to collect additional data on seasonal variations of herpetofauna in Belize. Herpetofauna that are of conservation concern in Belize (Table 5) still require continued monitoring and observations in order to target possible reasons for population decline and limiting factors for their distributions. Future research within Belize is important to provide data-based insight on herpetofauna species occurrences and the effects of anthropogenic change on their assemblages. Although there were some variations in reptile and amphibian species richness and diversity between habitats, our data shows the variations to be insignificant indicators of sensitivity towards anthropogenic changes in the study site. In fact, the Heavily Manicured Orchard was proportionate in herpetofauna species diversity and richness to the natural Lowland Broadleaf Forest, thus concluding that even constant anthropogenic activity had little effect on herpetofaunal assemblages in the area. The suspected reason for this lack of sensitivity is that each anthropogenically-altered habitat area had a surrounding natural forest edge, which could provide fauna with abundances of shelter and prey when necessary. Additionally, considering the significant alterations to standing vegetation and canopy percentages within each sampled habitat, the after effects of Hurricane Earl were minimal on herpetofaunal community composition within the study site. This data can be used to implement effective conservation methods by providing evidence that agricultural areas, when surrounded by natural habitat buffers, have little effect on herpetofaunal community assemblages.

**Table 5.** Herpetofauna species that occur in Belize considered as a concern for conservation (Critically Endangered = CE; Endangered = EN; Vulnerable = V; Near Threatened = NT; Lower Risk/Conservation Dependant = LR/CD).

REPTILES			
Species Name	Common Name	Conservation Status	Status Authority
*Dermatemys mawii*	Central American River Turtle	CE	Vogt et al. 2006
*Chelydra rossignonii*	Yucatan Snapping Turtle	V	Van Dijk et al. 2007a
*Rhinoclemmys areolata*	Furrowed Wood Turtle	NT	Van Dijk et al. 2007b
*Crocodylus moreletii*	Morlete’s Crocodile	LR/CD	Cedeño-Vázquez et al. 2012
*Crocodylus acutus*	American Crocodile	V	Ponce-Campos et al. 2012
*Celestus rozellae*	Rozella’s Lesser Galliwasp	NT	Sunyer et al. 2013a
*Phyllodactylus insularis*	Belize Leaf-tailed Gecko	V	Townsend and Walker 2014
*Agkistrodon bilineatus*	Cantil	NT	Lee and Hammerson 2007
**AMPHIBIANS**			
**Species Name**	**Common Name**	**Conservation Status**	**Status Authority**
*Lithobates juliani*	Maya Mountain Frog	NT	Lee and Walker 2004
*Smilisca cyanosticta*	Blue Spotted Mexican Treefrog	NT	Santos-Barrera et al. 2004a
*Incilius campbelli*	Campbell’s Forest Toad	NT	Lee et al. 2004a
*Craugastor laticeps*	Broadheaded Rainfrog	NT	Santos-Barrera et al. 2004b
*Craugastor chac*	Chac’s Rainfrog	NT	Walker et al. 2004
*Craugastor sandersoni*	Sanderson’s Streamfrog	EN	Lee et al. 2004b
*Craugastor sabrinus*	Long-legged Streamfrog	NT	IUCN SSC 2016
*Craugastor psephosypharus*	Limestone Rainfrog	V	Lee et al. 2004c
*Bolitoglossa dofleini*	Mushroom-tongue Salamander	NT	Cruz et al. 2010
*Eleutherodactylus leprus*	Leprus Chirping Frog	V	Santos-Barrera et al. 2004c
*Agalychnis moreletii*	Morelet’s Treefrog	CE	Santos-Barrera et al. 2004d

## Supplementary materials

Supplementary material 1 Common captures during study Data type: Occurrence Data Explanation note: Photos of reptiles and amphibians most commonly captured during the study. Information is provided regarding the habitats each species was caught in during the study, their IUCN Redlist conservation status, and EVS scores according to Johnson et al. (2015). This dataset is made available under the Open Database License (http://opendatacommons.org/licenses/odbl/1.0/). The Open Database License (ODbL) is a license agreement intended to allow users to freely share, modify, and use this Dataset while maintaining this same freedom for others, provided that the original source and author(s) are credited.Russell Gray, Colin T. Strine
