# Supplementary material for: Herpetofaunal assemblages of a lowland broadleaf forest, an overgrown orchard forest and a lime orchard in Stann Creek, Belize
Source: Zookeys. 2017 Oct 11;(707):131–65. doi: 10.3897/zookeys.707.14029 (PMC5674148; doi:10.3897/zookeys.707.14029)
Supplement: Supplementary material 1 — Common captures during study [file zookeys-707-131-s001.docx]

|  |
| --- |
| Appendix I |
| Common Captures during study |
|  |
|  |
|  |

| [Type the abstract of the document here. The abstract is typically a short summary of the contents of the document. Type the abstract of the document here. The abstract is typically a short summary of the contents of the document.] |
| --- |

*
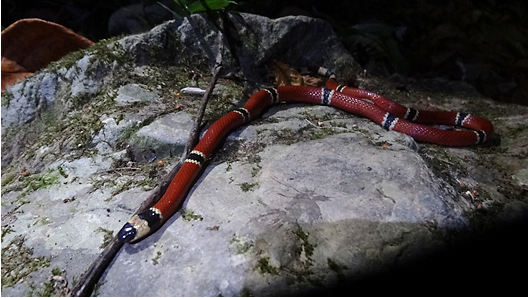
Micrurus hippocrepis* The Maya Coral Snake has been assessed by the IUCN Redlist as Least Concern (Acevedo, *et al.* 2013). This species has an EVS of 18; the lower end of the high-vulnerability category (Johnson *et al.* 2015). M. hippocrepis is a terrestrial, cryptozoic species, known to be an endemic to Central America, more specifically the Lowland Maya Forests and Moist Forests of Mesoamerica (Campbell, 1999; Radachowsky, 2002). During our study, this was the most commonly captured snake spececies (n=14) with occurences in the LBF, OGR, and HMO; LBF being the most abundant in captures. *Photo by Russell Gray*

*
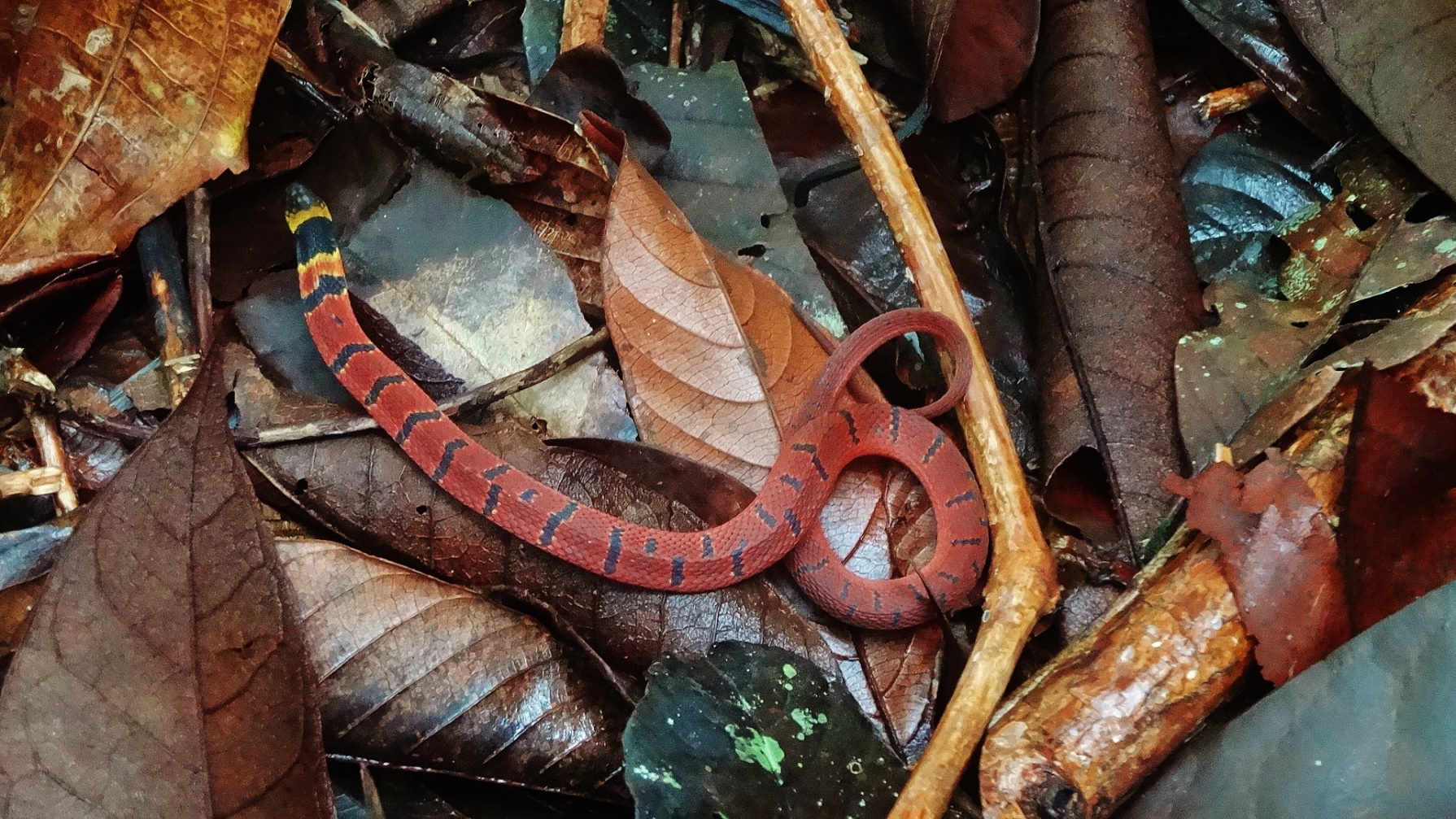
Ninia sebae* Redback Coffee are considered to be of Least Concern by the IUNC Redlist (Chaves, et al. 2013a). The species has a current EVS of 4, indicating a low end of the low-vulnerability category (Johnson, *et al.* 2015). *N. sebae* are small cryptozoic snakes that can be found in abundance in tropical forest areas (Greene, 1975). During this study, this was the second-most abundant snake species capture (n=9) and they were captured in all four habitat sites, the HMO accommodating the highest in abundance. *Photo by Russell Gray*


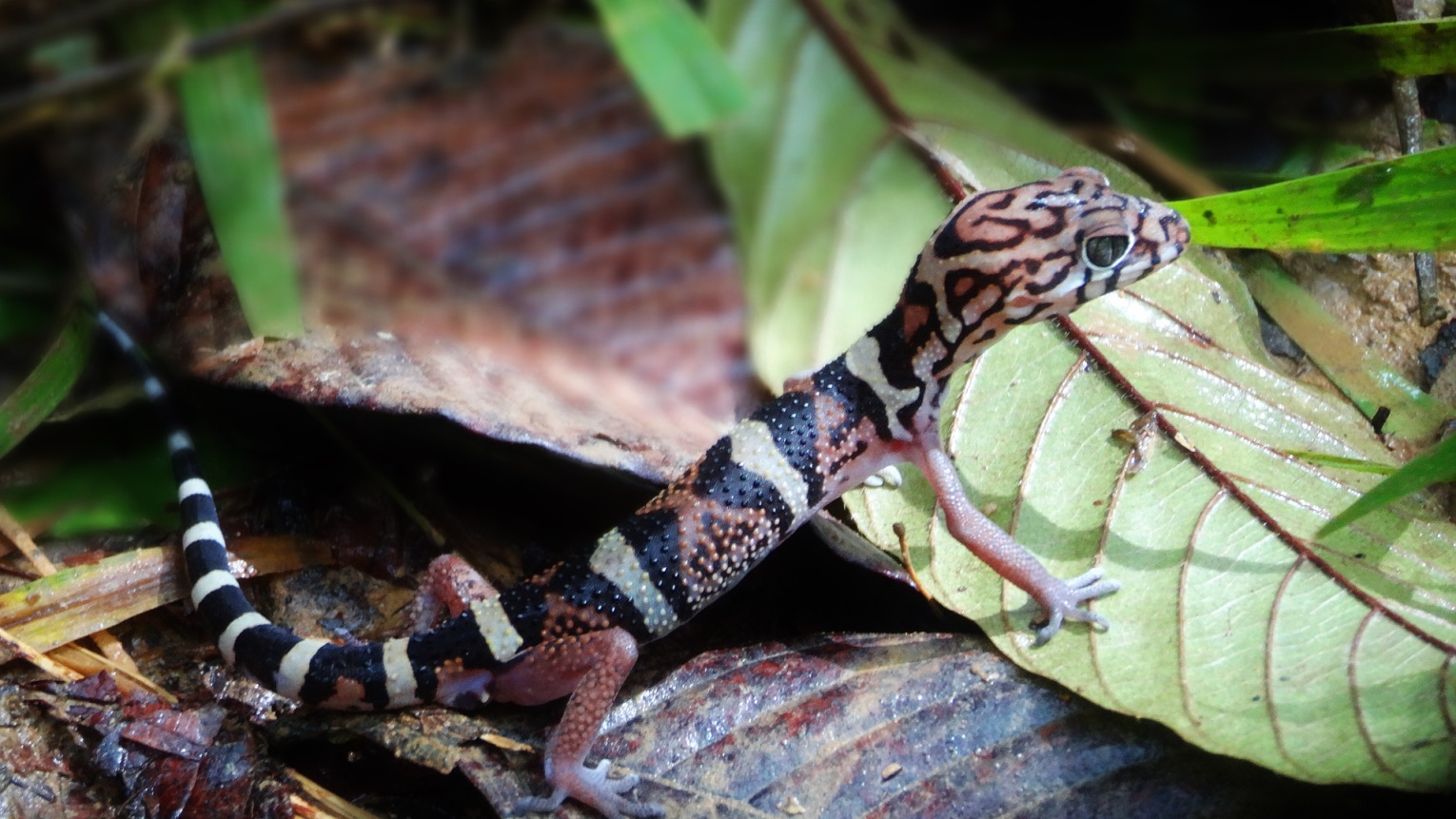


*Coleonyx elegans* the Yucatan Banded Gecko is considered to be of Least Concern by the IUNC Redlist (Sunyer, *et al*. 2013b). The EVS for C. elegans is currently 10; the lower end of the mid-range vulnerability category (Johnson, *et al*. 2015). As a terrestrial gecko, this species generally shelters under logs and rocks and can be found in a wide range of open and forested habitats throughout Central America (Campbell, 1999). During this study *C. elegans* was the fourth most abundant reptile capture (n=8). Captures occurred in the OGR, OGF and HMO, though no captures occurred in the LBF; the OGR was highest in capture abundance. *Photo by Russell Gray*

*
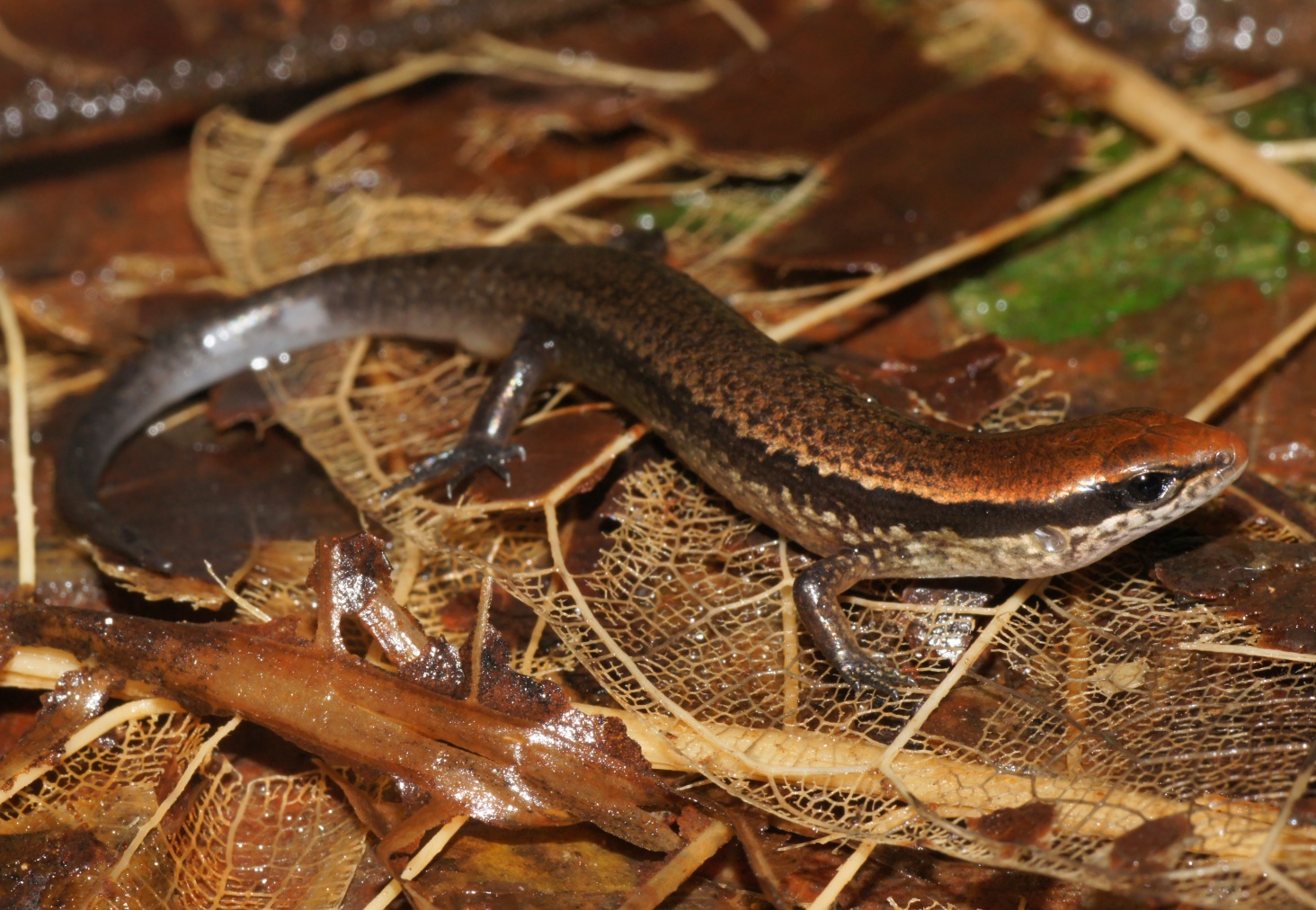
*

*Scincella cherriei* The Brown Forest Skink is considered to be of Least Concern by the IUNC Redlist (Chaves, *et al.* 2013b). This species has a calculated EVS of 7; the high end of the low-vulnerability category (Johnson, *et al*. 2015). S. cherriei is a terrestrial and cryptozoic skink which occurs in a wide range of moist and dry forested habitats across Central America (Campbell, 1999). During this study, this was the most abundant reptile along with *Micrurus hippocrepis* (n=14), occurring in all four habitat types with highest capture occurrences in the HMO. *Photo by César Barrio-Amorós/Doc Frog Photography*

*
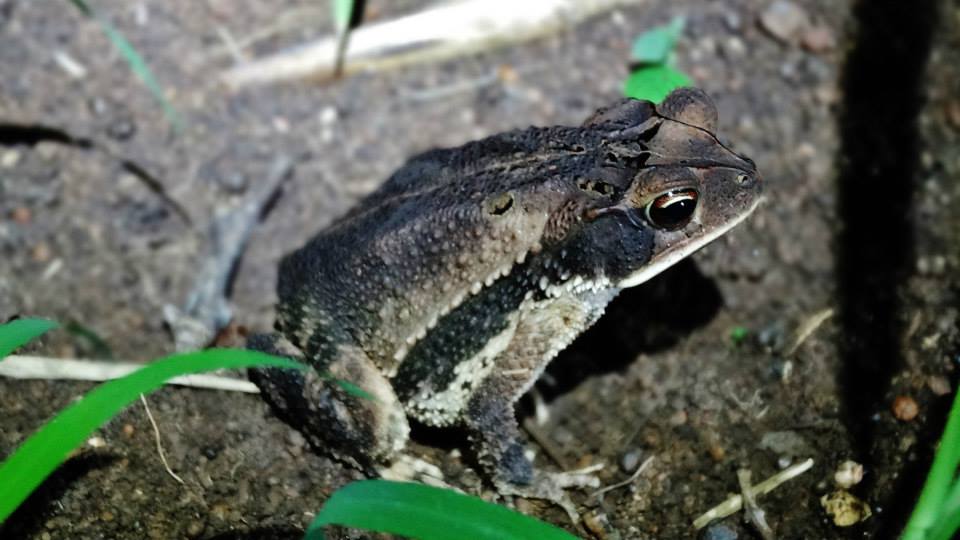
*

*Incilius valliceps* the Gulf Coast Toad is considered to be of Least Concern on the IUNC Redlist (Santos-Barrera, *et al.* 2010). The EVS for *I. valliceps* has been calculated as 6; the mid-range portion of the “Low-Vulnerability” category (Johnson *et al*. 2015). This toad can be found throughout a wide range of forested and non-forested habitat areas and is distributed ubiquitously from the Southern United States as far south as Nicaragua (Lee, 2000)*. I. valliceps* was the most prominent amphibian species captured during the study (n=37), with captures in all four habitats; the LBF having the highest quantity of capture, followed by the OGF. *Photo by Russell Gray.*

*
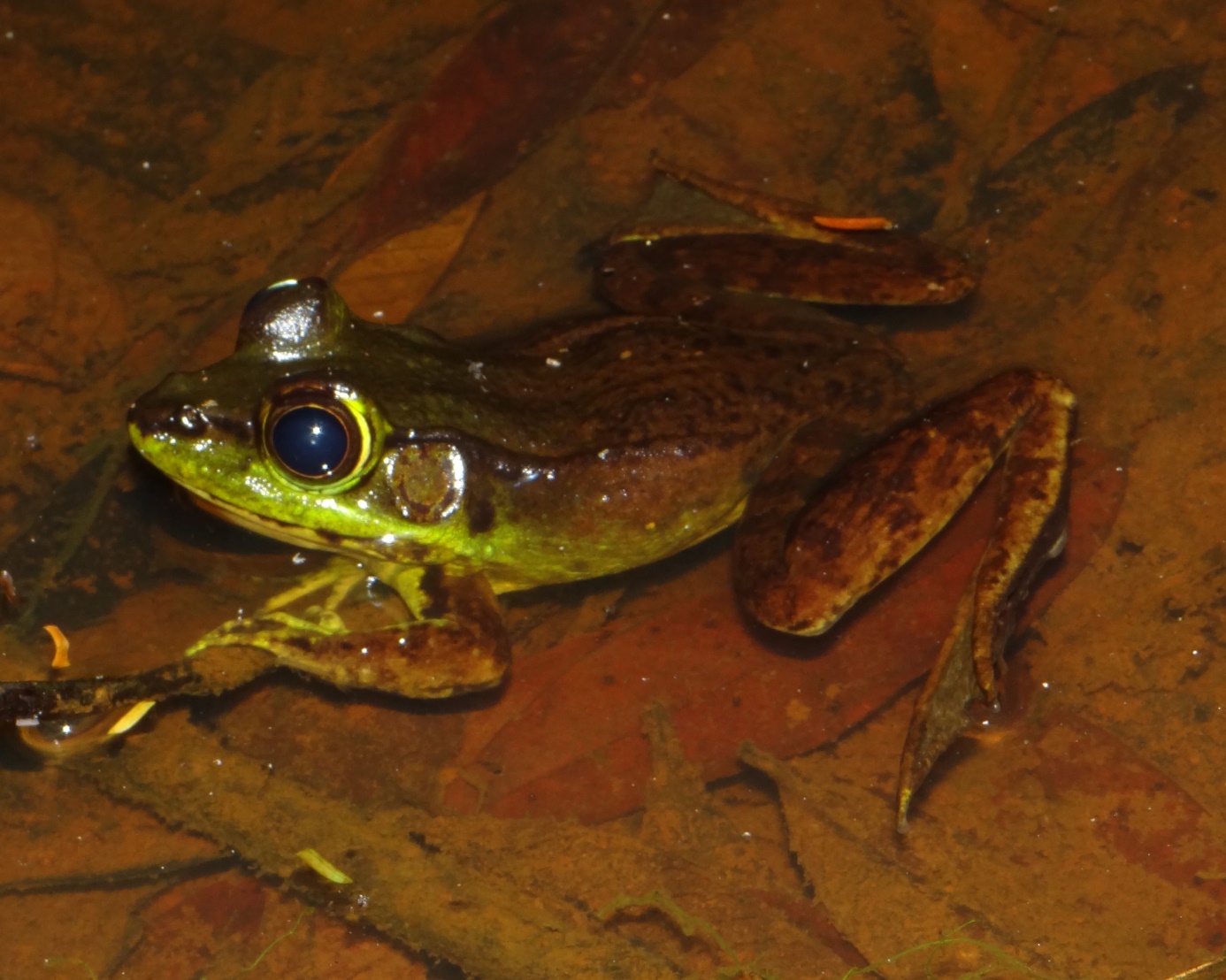
*

*Lithobates vaillanti* Vaillant’s Frog is considered to be of Least Concern by the IUCN Redlist (Santos-Barrera, *et al*. 2008). The species has been evaluated with an EVS of 9; the upper portion of the “Low Vulnerability” category (Johnson *et al.* 2015). This frog can be found in areas where there are permanent water bodies, and is distributed from Northern Central America down to parts of South America (Campbell, 1999). During this study, this was the second most frequent amphibian capture (n=33), with captures in the HMO, OGR and LBF; the HMO having the highest quantity of captures. *Photo by César Barrio-Amorós/Doc Frog Photography.*
